# The Enterics for Global Health (EFGH) *Shigella* Surveillance Study in Bangladesh

**DOI:** 10.1093/ofid/ofad653

**Published:** 2024-03-25

**Authors:** Farhana Khanam, Md Taufiqul Islam, Taufiqur Rahman Bhuiyan, Md Ismail Hossen, Md Nazmul Hasan Rajib, Shahinur Haque, Mahzabeen Ireen, Syed Qudrat-E-Khuda, Prasanta Kumar Biswas, Md Amirul Islam Bhuiyan, Kamrul Islam, Nazia Rahman, S M Azadul Alam Raz, Md Parvej Mosharraf, Md Elias Shawon Bhuiyan, Sadia Islam, Dilruba Ahmed, Faisal Ahmmed, Khalequ Zaman, John D Clemens, Firdausi Qadri

**Affiliations:** Infectious Diseases Division, International Centre for Diarrhoeal Disease Research, Bangladesh, Dhaka, Bangladesh; Infectious Diseases Division, International Centre for Diarrhoeal Disease Research, Bangladesh, Dhaka, Bangladesh; Infectious Diseases Division, International Centre for Diarrhoeal Disease Research, Bangladesh, Dhaka, Bangladesh; Infectious Diseases Division, International Centre for Diarrhoeal Disease Research, Bangladesh, Dhaka, Bangladesh; Infectious Diseases Division, International Centre for Diarrhoeal Disease Research, Bangladesh, Dhaka, Bangladesh; Infectious Diseases Division, International Centre for Diarrhoeal Disease Research, Bangladesh, Dhaka, Bangladesh; Infectious Diseases Division, International Centre for Diarrhoeal Disease Research, Bangladesh, Dhaka, Bangladesh; Infectious Diseases Division, International Centre for Diarrhoeal Disease Research, Bangladesh, Dhaka, Bangladesh; Infectious Diseases Division, International Centre for Diarrhoeal Disease Research, Bangladesh, Dhaka, Bangladesh; Infectious Diseases Division, International Centre for Diarrhoeal Disease Research, Bangladesh, Dhaka, Bangladesh; Infectious Diseases Division, International Centre for Diarrhoeal Disease Research, Bangladesh, Dhaka, Bangladesh; Infectious Diseases Division, International Centre for Diarrhoeal Disease Research, Bangladesh, Dhaka, Bangladesh; Infectious Diseases Division, International Centre for Diarrhoeal Disease Research, Bangladesh, Dhaka, Bangladesh; Infectious Diseases Division, International Centre for Diarrhoeal Disease Research, Bangladesh, Dhaka, Bangladesh; Infectious Diseases Division, International Centre for Diarrhoeal Disease Research, Bangladesh, Dhaka, Bangladesh; Infectious Diseases Division, International Centre for Diarrhoeal Disease Research, Bangladesh, Dhaka, Bangladesh; Infectious Diseases Division, International Centre for Diarrhoeal Disease Research, Bangladesh, Dhaka, Bangladesh; Infectious Diseases Division, International Centre for Diarrhoeal Disease Research, Bangladesh, Dhaka, Bangladesh; Infectious Diseases Division, International Centre for Diarrhoeal Disease Research, Bangladesh, Dhaka, Bangladesh; Director General Office, International Vaccine Institute, Seoul, Republic of Korea; Department of Epidemiology, Fielding School of Public Health, University of California, Los Angeles, Los Angeles, California, USA; Infectious Diseases Division, International Centre for Diarrhoeal Disease Research, Bangladesh, Dhaka, Bangladesh

**Keywords:** Bangladesh, disease surveillance, *Shigella* burden

## Abstract

**Background:**

*Shigella* is an important cause of diarrhea in Bangladeshi children <5 years of age, with an incidence rate of 4.6 per 100 person-years. However, the report was more than a decade old, and data on *Shigella* consequences are similarly outdated and heterogeneously collected.

**Methods:**

Facility-based disease surveillance is planned to be carried out under the Enterics for Global Health (EFGH) *Shigella* Surveillance Study consortium for 2 years with aims to optimize and standardize laboratory techniques and healthcare utilization and coverage survey, clinical and anthropometric data collection, safety monitoring and responsiveness, and other related activities. The EFGH is a cohesive network of multidisciplinary experts, capable of operating in concert to conduct the study to generate data that will pave the way for potential *Shigella* vaccine trials in settings with high disease burden. The study will be conducted within 7 country sites in Asia, Africa, and Latin America.

**Conclusions:**

We outline the features of the Bangladesh site as part of this multisite surveillance network to determine an updated incidence rate and document the consequences of *Shigella* diarrhea in children aged 6–35 months, which will help inform policymakers and to implement the future vaccine trials.

## Teaser

This article describes the Enterics for Global Health (EFGH) Bangladesh site, including the features of facility-based disease surveillance, population enumeration, healthcare utilization survey, and capacity-building initiatives for junior investigators for the EFGH study and future *Shigella* vaccine trials.

Shigellosis is an intestinal infection caused by *Shigella* bacteria. It is the most frequent cause of dysentery and a significant contributor to acute diarrhea [1]. Additionally, it has been associated with lower vaccination effectiveness, linear growth failure, and chronic diarrhea [2–4]. *Shigella* was identified as 1 of the 4 major pathogens in the Global Enteric Multicenter Study (GEMS) and Etiology, Risk Factors and Interactions of Enteric Infections and Malnutrition and the Consequences for Child Health and Development (MAL-ED) study conducted in Bangladesh and other countries [3, 5]. GEMS identified *Shigella* and pathogenic *Escherichia coli* as responsible pathogens for moderate to severe diarrhea (MSD) in children aged <5 years in Bangladesh [5]. In a molecular reanalysis study conducted in Bangladesh, *Shigella* was isolated from around 50% of invasive diarrhea, whereas culture detected only about 20% of them, indicating an underestimation of the *Shigella* burden due to the traditional culture method [6]. Despite being an endemic disease in low- and middle-income countries (LMICs) like Bangladesh, accurate and recent data on shigellosis-related morbidity and mortality are scarce [7]. Furthermore, multidrug-resistant *Shigella* has grown to be a significant threat to treatment and is often associated with serious complications, high treatment costs, and fatality [8]. Increasing rates of antibiotic-resistant *Shigella* and the high burden of *Shigella* infection in endemic settings show how urgent the need is for preventive measures such as vaccination intervention and improved infrastructure for safe water sources and sanitation.

Although there are a number of promising *Shigella* vaccines in development [9], phase 2b/3 *Shigella* vaccine trials will need to be conducted in areas where there is a high incidence rate of diarrhea caused by *Shigella*. To hasten the availability and acceptance of the vaccines among children, policymakers also need recent, country-specific data on the prevalence of *Shigella*-attributed diarrhea to understand the relative importance of this vaccine-preventable disease.

The Enterics for Global Health (EFGH) consortium established a *Shigella* disease surveillance network accompanying clinical and laboratory preparation within 7 country sites in Asia, Africa, and Latin America (Bangladesh, Kenya, Malawi, Mali, Pakistan, Peru, and The Gambia) to support the upcoming *Shigella* vaccine trials in settings with a high disease burden. This article describes the structure and characteristics of Bangladesh site to carry out facility-based disease surveillance to establish the incidence rate of *Shigella* in children aged 6–35 months.

## SITE DESCRIPTION

The EFGH Bangladesh site is located in a densely populated urban area of Dhaka city, the capital city of Bangladesh. Bangladesh is situated between latitudes 20°34′N and 26°38′N and longitudes 88°01′E and 92°41′E in the northeastern region of South Asia. It covers more than 147 570 square kilometers (56 977 square miles) in total. The great Himalayan Range and the Bay of Bengal are located to the north and south, respectively, of the country [10]. Bangladesh is the eighth-most populated country, comprising 2.2% of the world's population [11]. In 2022, the total population was estimated to be 169 828 911, with a density of 1119 people per square kilometer [10]. According to the World Population Review, Bangladesh ranks ninth in the world in terms of population density [12]. The numbers of males and females were 84 077 203 (49.51%) and 85 653 120 (50.43%), respectively, with 16 263 550 (9.58%) children <5 years of age [10]. With a median age of 26 years, Bangladesh's population has a very even age distribution, and a significant portion of the entire population is made up of young people aged 15–29 years [13]. The weather in Bangladesh is tropical monsoon, with 3 distinct seasons: winter (November to February), premonsoon (March to May), and monsoon (June to October). Bangladesh often experiences warm weather throughout the year with average temperature of 20°C–35°C (68°F–95°F). The rainy season, which lasts from April to September, coincides with the warmest months, while the winter is cooler and drier. High amounts (80% or more) of humidity are present, especially during the monsoon season. The average annual rainfall in Bangladesh is roughly 2200 mm; most regions receive at least 1500 mm, while some other regions, such the northeastern border, receive about 5000 mm. Bangladesh sees high rainfall during the monsoon season [14]. The prevalence of diarrheal diseases in Bangladesh changes with the seasonal variations [15]. The number of diarrheal patients increases during high and low rainfall. Higher temperatures led to an increase in diarrheal cases, especially among individuals with lower socioeconomic and sanitation status [16]. The flood-affected area in Bangladesh provides a clear predictor for the number of diarrheal cases [17].

The current literacy rate is 74.66% among the population aged ≥7 years [10]. The average maternal education level has increased from 2004 to 2018, and a 62% gross reduction in the no education group and a 61% gross increase in the higher education group were seen during this period [18]. The per capita gross domestic product (GDP) of Bangladesh is US$2458, according to the World Bank report for 2021 [19]. More than half of Bangladesh's GDP is generated by the services sector, which is the primary economic activity in the nation. The industry sector, which accounts for around 28% of GDP, is the second-largest economic activity. With a 15% GDP share, the agriculture industry is the third-largest economic activity [13]. The current rate of unemployment is 3.51%, according to the report published by the Planning Commission in 2023 [20]. Around 40% of the population has access to all 3 major water, sanitation, and hygiene (WASH) components. There is a significant difference in WASH coverage between urban and rural areas; a higher (51.6%) prevalence of combined WASH facilities is available in urban areas compared to rural areas (36.9%) [21]. More than 97% of the population used groundwater for their drinking water supply in rural areas, whereas 82% of the water supply is extracted from groundwater in urban Dhaka. The remaining 18% relies on 3 surface water treatment plants in Dhaka city [22]. According to the current Bangladesh Bureau of Statistics report, 56.04% of people of in Bangladesh use a latrine with safe disposal by flushing or pouring water [10]. Studies conducted in Bangladesh have shown that 23% of children <5 years of age and one-third of ever-married women are underweight and 28% of children <5 years of age are stunted [23]. Around 13% of underweight women have short stature, which eventually acts as a risk factor for difficult childbirth and low-birth-weight infants [24].

## HEALTHCARE SYSTEMS AND IMMUNIZATION SCHEDULE

Bangladesh has a 3-tiered, decentralized healthcare system that is supervised and controlled by the government, for-profit businesses, nongovernmental organizations (NGOs), and local and foreign charitable organizations. The government is the main healthcare provider responsible for providing comprehensive health services through the primary (Upazila Health Complexes, Union Health and Family Welfare Centres, Mother and Child Welfare Centres, and community clinics at ward level), secondary (district hospitals, general hospitals, and medical colleges), and tertiary (teaching and specialized hospitals) healthcare services [25, 26]. The public sector provides curative, preventive, promotive, and rehabilitative services. The private sector provides curative services while NGOs, on the other hand, mainly provide preventive and basic care [27].

Immunization is a highest-priority program of Bangladesh and every year >3.7 million target children receive at least 5 vaccines composed of 11 antigens [28]. Bangladesh has achieved and maintained high routine immunization coverage, though the coverage was not uniformly distributed across the geographical area, mother's education and wealth. The government, through its annual Expanded Programme on Immunization (EPI) [29] work plan, has developed strategies to address the challenges that exist in achieving high coverage. The human papillomavirus vaccine will be included in EPI in 2023. The typhoid conjugate vaccine and Japanese encephalitis vaccine will also be introduced gradually in Bangladesh. Bangladesh is expected to formally graduate from least developed country status in 2026, after a 5-year transition period that has caused constraints in accessing funds from Gavi, the Vaccine Alliance to support the routine immunization program [30]. However, Gavi recently announced that it would support the national immunization program till 2029 at the request of the Bangladesh Parliamentary Forum for Health and Wellbeing. The routine immunization schedule in Bangladesh for childhood vaccines is shown in Table 1 [31].

**Table 1.** Expanded Programme on Immunization Schedule in Bangladesh [25]

Name of Disease	Name of Vaccine	No. of Doses	Interval Between Doses	Earliest Age of Vaccination
Childhood tuberculosis	BCG	1	…	At birth
Diphtheria, pertussis, tetanus, hepatitis B, *Haemophilus influenzae* type b	HibPenta	3	4 wk	6 wk 10 wk 14 wk
Poliomyelitis	bOPV	3	4 wk	6 wk 10 wk 14 wk
Poliomyelitis	fIPV	2	8 wk	6 wk 14 wk
Pneumococcal pneumonia	PCV10	3	4 wk	6 wk 10 wk 14 wk
Measles and rubella	MR	2	6 mo	9 mo 15 mo

Abbreviations: bOPV, bivalent oral poliovirus vaccine; fIPV, fractional-dose inactivated polio vaccine; MR, measles-rubella; PCV10, 10-valent pneumococcal conjugate vaccine.

## HISTORICAL *SHIGELLA* INCIDENCE, PREVALENCE, AND ANTIMICROBIAL RESISTANCE DATA OF BANGLADESH

The GEMS conducted in Bangladesh between 2007 and 2011 found incidence rates of MSD attributable to *Shigella* of 1.7, 8.5, and 3.1 cases per 100 child-years for children aged 0–11 months, 12–23 months, and 24–59 months, respectively [5]. *Shigella* was identified as 1 of the top 4 pathogens in this study, and the isolation rates of *Shigella flexneri*, *Shigella sonnei*, *Shigella boydii*, and *Shigella dysenteriae* were 67.9%, 24.4%, 3.9%, and 3.8%, respectively. A total of 89.4% *S flexneri* was made up of 5 serotypes or subserotypes: *S flexneri* 2a, *S flexneri* 6, *S flexneri* 3a, *S flexneri* 2b, and *S flexneri* 1b [32]. A multicenter study of *Shigella* diarrhea conducted in Bangladesh between 2000 and 2004 showed that the overall incidence of treated shigellosis was 2.1 episodes per 1000 persons per year in all ages and the rate was significantly higher in Bangladesh than other study sites [33]. The rate was 46.1 per 1000 persons per year for children <5 years of age [33]. Another study conducted in Bangladesh has shown that *Shigella* was significantly higher in rural areas than in urban sites [34]. A metanalysis of the updated and comprehensive assessment of the shigellosis drug resistance burden in Bangladesh demonstrated a higher prevalence rate for multidrug-resistant *Shigella* species, and the resistance rates were 61.9% for fluoroquinolone, 60.8% for trimethoprim-sulfamethoxazole, 38.8% for azithromycin, 36.2% for nalidixic acid, 34.5% for ampicillin, and 31.1% for ciprofloxacin [35].

## HISTORICAL HEALTHCARE-SEEKING BEHAVIOR FOR CHILDREN WITH DIARRHEA IN BANGLADESH

A study on healthcare-seeking behavior, conducted in a high-risk population residing in Mirpur, Dhaka, Bangladesh, between April and September of 2010, reported that only 6% of patients with MSD visited a healthcare facility. Most of the patients with mild illness did not seek treatment and were likely resolved with self-treatment such as oral rehydration solution (ORS). The study also suggested that there are several factors that influence the care-seeking behavior of children with diarrheal diseases including age, sex, educational level, living condition, socioeconomic status, and distance to healthcare facilities [36]. The study also suggested that caregivers of the children with diarrheal illnesses were more likely to seek care than older individuals in the event of severe diarrhea [36]. Studies also report that most caregivers seek care initially from nonprofessional healthcare providers, such as drug sellers or traditional healers, and only a small proportion of children receive appropriate treatment, such as oral rehydration therapy or zinc supplementation [36]. A study conducted in a rural area of Mirzapur, Bangladesh, found that 87.9% of children with diarrhea sought treatment outside of their homes: 22.1% received treatment from a licensed healthcare provider, 5.5% visited a private doctor, and the majority purchased medicines from pharmacies or shops [37]. The Centre for International Epidemiological Training, Canada, conducted a survey in Bangladesh in 2003 that showed 13% of individuals seek care from government services, 27% use private or NGO services, and 60% visit unqualified services [25]. To encourage people to use healthcare systems, the Ministry of Health and Family Welfare, Government of the People's Republic of Bangladesh, has implemented a number of efforts, including offering free or inexpensive services, increasing immunization coverage, boosting maternal and child health, and working with NGOs and donor organizations [38]. The healthcare system in Bangladesh still faces a number of obstacles and inadequacies, including poor infrastructure, a lack of human resources, poor care quality, and unequal access [39]. As part of its efforts to improve the healthcare system, the government is also putting out reforms and policies aimed at strengthen primary healthcare, boosting health funding, assuring universal health coverage, and addressing new health problems [40].

## LABORATORY FACILITIES IN ICDDR,B

The Clinical Microbiology Laboratory (CML) under the Laboratory Sciences and Services Division at the International Centre for Diarrhoeal Disease Research, Bangladesh (icddr,b) carries out microbiological and antimicrobial analysis of specimens from outpatients as well as for projects following Good Clinical Laboratory Practice (GCLP) guidelines and International Organization for Standardization (ISO) 15189 requirements. The microbiological laboratory for enteric pathogens are Biosafety Level 2, with internal and external quality assurance. The CML has long experience of using a quality management system with the credibility of achieving the ISO 15189 accreditation for icddr,b with updates carried out every 2 years. The laboratory also follows the international External Quality Assurance System program and organizes in-country interlaboratory comparison programs. A laboratory information management system is being used to manage laboratory data, streamline workflows, and improve data quality. The team members have long-term experience in culturing, biochemical testing, and serotyping to identify the specific isolates of different enteric pathogens, such as *Shigella*, *Vibrio cholerae*, *Salmonella*, enterotoxigenic *Escherichia coli*, and others. There are facilities for carrying out antimicrobial susceptibility testing by disk diffusion method, and interpreted based on the Clinical and Laboratory Standards Institute guidelines. The minimum inhibitory concentration of the antibiotics is also determined. VITEK 2, an automated antimicrobial susceptibility testing system, is also used in the CML and provides identification and susceptibility results in as little as 5 hours. The Mucosal Immunology and Vaccinology Laboratory (MIVL) under the Infectious Diseases Division at icddr,b is equipped with facilities for analysis of stool and blood specimens for study-related purposes. All techniques in the MIVL are carried out following the guidelines of GCLP and according to the study-specific standardized operating procedures. The MIVL laboratory has several biohazard safety hoods for the processing of biological samples and for maintaining sterile conditions for specimen processing. For the fractionation of samples, refrigerated tabletop centrifuges, high-speed centrifuges (Beckman), and ultracentrifuges (Beckman L7-80 and a Beckman L5-65B) are also available. Incubators are present for carrying out the conventional microbiologic assays of stool and rectal swab. Equipment and expertise are available for simple polymerase chain reaction (PCR) techniques and also for multiplex quantitative PCR and TaqMan Array Card. There are multiple ultra-low-temperature freezers, −20°C freezers, refrigerators, and liquid nitrogen dewars for specimen storage with a power backup generator. Additionally, there is a special flow cytometer laboratory within the facility, which has FACSCalibur as well as FACSAria III to detect 9 fluorochromes (this capacity is expandable if needed), as well as a 96-well plate adapter for single cell sorting. Both CML and MIVL are now working with the EFGH project with the goal of providing baseline data in advance of the *Shigella* vaccine trial.

## EFGH CATCHMENT AREA AND RECRUITMENT FACILITIES

**Figure 1.**
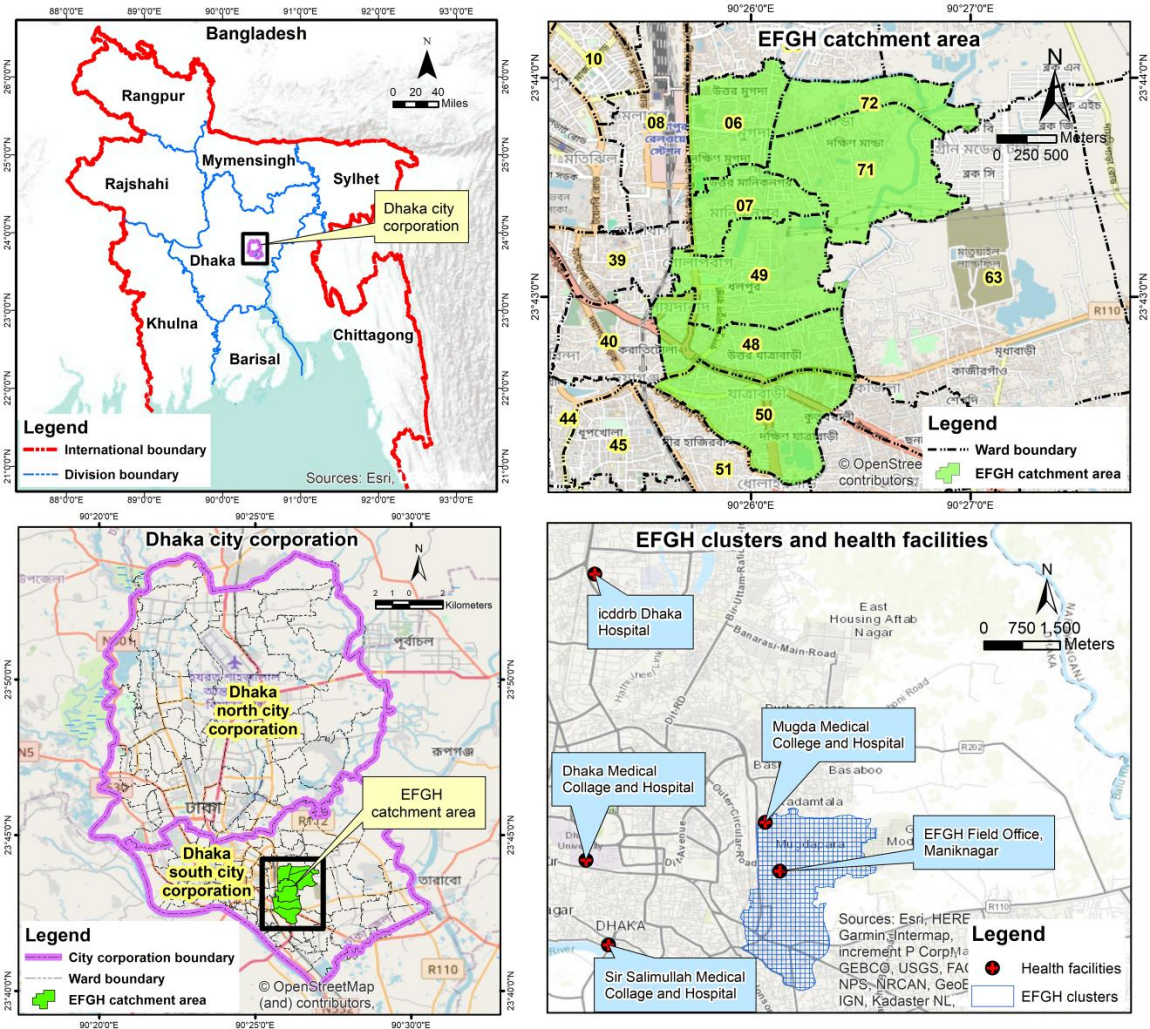
Map of Enterics for Global Health (EFGH) catchment area and healthcare facilities of the Bangladesh site. We have prepared the figures by using the licensed version of the Software ArcGIS10.8.1 (ESRI Inc.) Background map: esri open source map, Bangladesh map: Humanitarian data exchange (HDX)admin map.

The EFGH study catchment area includes 7 wards (wards 6, 7, 48, 49, 50, 71, and 72) of Dhaka South City Corporation, covering an approximate population of 300 000 persons. The geographic location of the study catchment area is between 23°42′7.09′N and 23°44′1.06′N and 90°25′32.90′E and 90°27′2.94′E (Figure 1). The icddr,b Dhaka Hospital, EFGH Field Office, Dhaka Medical College and Hospital (DMCH), Mugda Medical College and Hospital (MMCH), and Sir Salimullah Medical College and Hospital (SSMCH) are among the healthcare facilities from which patients are enrolled for the disease surveillance. The recruitment facilities in the study catchment area were selected based on the most preferred responses by caretakers during a short survey conducted in the catchment area about seeking care for their child's diarrheal diseases. The icddr,b Dhaka Hospital provides a broad spectrum of services, particularly for diarrheal diseases, with infants and children representing a high proportion of patients. Each year, it treats >140 000 patients with diarrhea. Dhaka Hospital offers an infrastructure for a wide range of clinical research projects in addition to treating patients with diarrhea. Additionally, it plays a critical role in fields like disease surveillance, monitoring for antimicrobial resistance, and clinical training. The EFGH Field Office is newly established field office, located within the same neighborhood as the study population. It is a dedicated, project-based field site and is under the central security system of the icddr,b main campus, with internet facilities for data entry and transfer. DMCH, MMCH, and SSMCH are tertiary-level hospitals with services including emergency, outpatient, inpatient, and investigation facilities for 24 hours. In DMCH, MMCH, and SSMCH, there are roughly 2300, 3500, and 900 beds available to treat admitted patients, respectively. The EFGH staff have been integrated into all of these facilities where they strategically work to enroll children between 6 and 35 months of age from the EFGH catchment population when prescreening and screening procedures are complete, as well as to attend to all children aged <5 years who seek treatment with diarrhea there. In all recruitment facilities, patients are treated for free. However, to supplement the government's supplies in times of scarcity, the EFGH consortium provides zinc, ORS, intravenous fluids, and antibiotics. The culture and antibiotic susceptibility results for *Shigella* isolated from specimens from enrolled patients are conveyed back to the same facility as part of the commitment to the facilities, helping to direct ongoing clinical management of the specific patients.

## CLINICAL MANAGEMENT AND GUIDELINE ADHERENCE

The EFGH study team provides clinical management of study participants based on the diarrheal treatment guidelines of the treatment facilities. The World Health Organization diarrheal treatment guidelines are followed by the tertiary hospitals for treating the patients [41, 42]. In the icddr,b Dhaka Hospital and EFGH field office, patients are treated by following the icddr,b's treatment guideline. In the icddr,b guideline, severe dehydration is managed with intravenous fluid (preferable Ringer's lactate or cholera solution [100 mg/kg]) immediately after admission to the hospital, followed by ORS (5 mL/kg/hour) as soon as the child is able to drink. Patients with some dehydration are managed by ORS over 4 hours. In case of no dehydration, ORS is prescribed. Antibiotic administration usually starts with azithromycin (10 mg/kg for 5 days), and if there is no improvement after 48 hours, pivmecillinam (15 mg/kg every 6 hours) is started and continued for 5 days. The study team ensures zinc supplementation to every child for 10–14 days regardless of dehydration status. In the EFGH field office, patients with no and some dehydration are managed; however, patients with severe dehydration are referred to the icddr,b Dhaka Hospital for better management. The detailed treatment guideline for diarrhea management in healthcare facilities is shown in Table 2.

**Table 2.** Current Treatment Guidelines for Diarrheal Management

Category or Population	Country or Site-Specific Guideline
**Dehydration management (without severe acute malnutrition)**	
Severe	Guideline followed by all EFGH health facilities Plan C: Start IV fluid immediately (preferably Ringer’s lactate/cholera solution [100 mL/kg]) Reassess every 15–30 min Give ORS (5 mL/kg/h) as soon as the child can drink Reclassify dehydration after 6 h in infant & 3 h in child and continue with A, B, C plan
Some	Guideline followed by all EFGH health facilities Plan B: Give recommended ORS in clinic over 4 h Reclassify dehydration after 4 h and continue with A, B, C plan
None	Guideline followed by all EFGH health facilities Plan A: Increase food and fluid intake to prevent dehydration
**Dehydration management (with severe acute malnutrition and no shock)**	
Severe	Guideline followed by the icddr,b Dhaka Hospital If the child has some dehydration, ORS is given 10 mL/kg/h for the first 2 h, then 5 mL/kg/h for next 10 h or until the fluid deficit is corrected. In addition, ongoing stool losses are replaced with 5–10 mL/kg of ORS after each watery stool. The mother is advised about the quantity of ORS to be given, in terms of teaspoons per hour (eg, 40 mL/h = 8 teaspoons/h). If a child is unable to drink due to weakness or vomiting, ORS is administered through a NG tube drip. If the child has some dehydration but requires IV fluids because of vomiting that continues even after introducing a NG tube, or because of severe abdominal distension, IV half strength cholera saline with 5% dextrose is given 10 mL/kg/h for the first 2 h, then 5 mL/kg/h till the fluid deficit is corrected, or when the child is able to take ORS. If possible, extra potassium is added to the infusion (injection potassium chloride, 13 mmol/L of infusion). For children with severe dehydration, initial hydration is done with IV cholera saline with 5% dextrose. If possible, 7 mmol of potassium is added to each liter of infusion. Vitamin A, 200 000 units, is given to children >1 y of age, 100 000 units for infants 6–12 mo, and 50 000 units for those <6 months of age. Folic acid, 1.25 mg, is given once daily at least for 1 mo. Multivitamin drops are provided twice daily for at least 1 mo, 1 mL to children >1 y of age. Potassium is given orally in a dose of 4 mmol/kg/d, 2–3 times daily, for 5 d. Injection magnesium sulphate (50%), 0.1 mL/kg (0.4 mmol/kg), is given IM once daily for 7 d. Guideline followed by the other health facilities 5–10 mL/kg/h for the next 4–10 h on alternate hours with F75
Some	Guideline followed by the icddr,b Dhaka Hospital and other health facilities Same as above
None	Not applicable
**Therapeutic zinc**	
All children	Guideline followed by all EFGH health facilities Zinc supplementation for 10–14 d (age ≤6 mo: 10 mg/d; age >6 mo: 20 mg/d)
**Antibiotics**	
Dysentery or *Shigella* upon culture confirmation	Guideline followed by the icddr,b Dhaka Hospital Syrup azithromycin: 10 mg/kg once daily for 5 d. If there is no improvement within 48 h with azithromycin, stop azithromycin and start pivmecillinam 15 mg/kg per dose every 6 h for 5 d. (If the patient is receiving ampicillin or ciprofloxacin, please continue this as the treatment of invasive diarrhea and observe for 48 h. If there is deterioration within 24 h or does not improve within 48 h, azithromycin should be started. However, if the patient receives ceftriaxone for another indication, continue it at least for 3 d and there is no need to add azithromycin.) Guideline followed by the other health facilities Ciprofloxacin (15 mg/kg) twice daily for 3 d OR based on local sensitivity IV/IM ceftriaxone at 50–80 mg/kg/d for 3 d (if child is severely ill or as second-line treatment) Other second-line therapies (azithromycin, cefixime, TMP-SMX)
Suspected cholera (age ≥2 y + severe dehydration + cholera present in area)	Guideline followed by the icddr,b Dhaka Hospital Use of doxycycline (single dose) for the treatment of suspected or proven cholera: Age ≥18 y in males and nonpregnant females: capsule doxycycline 300 mg Age 11–17 y (weight <50 kg): capsule doxycycline 200 mg^a^ Age 5–10 y (if patient can swallow): capsule doxycycline 100 mg (2–4 mg/kg single dose) Azithromycin will be used in the following cases only: pregnant females (tablet azithromycin 1 g) Age <5 y: syrup azithromycin 20 mg/kg Guideline followed by the other health facilities Erythromycin (12 mg/kg) 4 times a day for 3 d Ciprofloxacin 10–20 mg/kg twice per day for 5 d TMP-SMX: 4 mg/kg trimethoprim and 20 mg/kg sulfamethoxazole twice a day

Abbreviations: EFGH, Enterics for Global Health; icddr,b, International Centre for Diarrhoeal Disease Research, Bangladesh; IM, intramuscular; IV, intravenous; NG, nasogastric; ORS, oral rehydration solution; TMP-SMX, trimethoprim-sulfamethoxazole.

^a^If the weight is ≥50 kg, then the dosage will be 300 mg.

The study team encounters a variety of challenges while providing diarrheal management, such as patients frequently not finishing their antibiotic courses and taking antibiotics before enrollment, patients missing appointments for follow-up care, tertiary-level hospitals occasionally running out of medications, and patients being unable to purchase prescribed medications. However, the EFGH team helps to reduce these challenges by providing as many resources as possible.

## TRAINING AND CAPACITY BUILDING

The EFGH Bangladesh leadership promotes the advancement of young researchers. The EFGH consortium leadership supported 4 online courses including Introduction to Epidemiology for Global Health, Fundamentals of Global Health Research and Leadership, Economic Evaluation in Global Health, and Management in Health from the University of Washington for 12 different investigators to complete in order to achieve this objective. The team members obtained the knowledge and expertise from the training they needed to carry out their responsibilities and achieve the goals of the study. Two junior investigators attended the annual investigators meeting in Seattle, Washington, USA. Two applications were submitted from the Bangladesh site for the EFGH Rising Star award, of which 1 application was funded by the consortium. Two investigators were selected to lead the 2 EFGH substudies (inflammatory biomarker and EFGH serologic studies). Four investigators attended the training-of-trainers in Kenya to conduct the down-training for the Bangladesh site. A long-lasting research collaboration has been established with several institutions through the EFGH study.

## ETHICAL CONSIDERATIONS

Prior to study initiation, study materials were approved by the Institutional Review Board, comprised of the Research Review Committee and Ethical Review Committee at the icddr,b. This study is being carried out according to Good Clinical Practice, including GCLP, the Declaration of Helsinki, IRBs, and local rules and regulations.

## CONCLUSIONS

The study will provide insights on the updated disease burden of *Shigella*-attributed diarrhea, which will be crucial for decision-making on vaccine trials in EFGH sites with high burden. The study will document the consequences of *Shigella* diarrhea, the healthcare utilization pattern, the cost of illness, and the current magnitude of antimicrobial resistance among Bangladeshi children. The findings will also accelerate the implementation for potential vaccines to prevent shigellosis among children in LMICs like Bangladesh.

## Notes

***Acknowledgments.*** The icddr,b is grateful to the governments of Bangladesh and Canada for providing core/unrestricted support. We thank the study participants in advance; all study staff for supporting the study; and the physicians, administration, and health officials at each EFGH recruitment site who are kindly providing facilities and support to carry out the study. We are grateful to the EFGH Coordination Team in helping us set up and carry out the study activities with their continuous and kind support.

***Supplement sponsorship.*** This article appears as part of the supplement “Enterics for Global Health (EFGH) *Shigella* Surveillance Study-Rationale and Methods,” sponsored by the Bill & Melinda Gates Foundation.

***Financial support.*** This work was supported by the Bill & Melinda Gates Foundation (grant numbers INV-016650, INV-036891, INV-028721, and INV-041730).
